# Eclectic Review on Criminal Abortion

**Published:** 1830-07-01

**Authors:** 


### XIII.

### RETROSPECTIVE REVIEW.

### CRIMINAL ABORTION.*

*Sed jacet aurato vix ulla puerpera lecto,*

*Tantùm artes hujus, tantum medicamina possunt,*

*Quæ steriles facit, atque homines in ventre necandos*

*Conducit.* JUVENAL.

*Vestra quid effoditis subjectis viscera telis,*

*Et nondum natis dura venena datis?*

*Quæ prima instituit teneros convellere foetus*

*Malitiâ fuerat digna perire suâ.*

*Hoc neque in Armeniis tigres fecère latebris,*

*Perdere nec foetus ausa leœna suos.*

*At teneræ faciunt, sed non impunè puellæ,*

*Sæpè suos quæ utero necat, ipsa perit.* OVID.

*Definition of the crime.* The employment of any means intended to destroy the fœtus *in utero*, or to procure its expulsion previously to the period at which it is capable of existing, independent of its connexions with the mother, constitutes the crime of provoked abortion. This definition obviously excludes the abortion consequent on the employment of medicine, or infliction of violence, taken or incurred without criminal design against the offspring.

Abortion may be involuntary, as induced by the operation of natural causes, or premeditated and determined by external violence. It is the province of the juridical physician to decide whether, in any suspected case, the event has taken place accidentally, or been the consequence of criminal proceeding; whether, and by what means, the fœtus has been prematurely expelled; and, lastly, whether, at the period of employment of such means, the fœtus were endued with life.

But previously to our entering upon an exposition of the general principles by which the solution of these important queries may be directed, it may be right to consider the causes by which abortion, spontaneous or voluntary, is commonly produced, and the external and internal signs from which its occurrence may be very confidently inferred.

Spontaneous or involuntary abortion may arise from causes operating directly either on the mother, or the fœtus and its appendages. Under the first class of causes may be enumerated peculiarity of constitution; moral affections, particularly grief† and the other depressing passions, surprize, terror, sympathy, sometimes excited by the view of another woman in labour; agents which exert a physical operation, as undue plethora, weak-

* Dict. des Sciences Medicales, &c.

† This will be particularly liable to operate on an unfortunate female about to give birth to an illegitimate child, and whose mind is keenly alive to the shame and wretchedness of her situation. EDIT.

ness, and irritability; inordinate exertion of any kind, excessive evacuations, intemperance, acute and violent diseases, especially those of the uterus or organs composing the generative and urinary systems, or mechanical injury. Maladies of the fœtus or a morbid condition of the funis, the membranes, or placenta, belong to the second class.

The means by which artificial or voluntary abortion may be determined are either constitutional, as exciting a general disturbance in the economy; or local, and exerting a mechanical operation. The first are by far the least certain in their effects. They consist principally of blood-letting, evacuations from the mouth and rectum, diuretics and emmenagogues. We shall cursorily consider these different agents, and the rank which they respectively hold in the catalogue of abortives.

*Blood-letting*. *Mulier, in utero ferens, sectâ venâ abortit*, is one of the aphorisms of the venerable Hippocrates: and a similar opinion generally prevails among the people. But abstraction of blood, although when largely employed in a weak, delicate, and irritable female, or otherwise injudiciously resorted to, may operate in provoking abortion; under other circumstances, and especially in plethoric subjects, is often successfully prescribed to obviate the impending danger. Copious bleeding from the *foot* in the earlier stages of pregnancy is commonly considered more likely to induce disastrous consequences than the same operation performed in the arm, and at a more advanced period of utero-gestation.

*Emetics and Purgatives*. These act either by the general commotion which they excite in the system, or the irritation produced by them in the intestinal canal, and hence propagated to the hypogastric organs. Yet a vomit or aperient, seasonably administered, may remove intestinal disorder, which, otherwise, might have eventually determined abortion. When gastric or alvine evacuants are administered with this view, those of the mildest operation should be selected, and great precaution exercised in their employment.* They, on the contrary, who use such remedies with criminal intention, most commonly choose such as are distinguished by the violence of their action; and take, or administer them in immoderate doses, independently of all necessity or indication.

*Diuretics and Emmenagogues*. Various medicinal substances, which act directly on the urinary organs, are regarded as capable of provoking the menstrual flux, and consequently abortion. But it may be doubted, whether any diuretic or emmenagogue, with the exception of the *lytta*, really possesses any such baneful property. Preparations of savin, and the terebinthinates, have often been very largely administered without inducing these consequences. Electricity, when applied in considerable force, may, particularly in subjects otherwise predisposed to abortion, excite a disturbance in the uterine system, destructive to the healthy performance of its gestatory functions. This will belong to the class of constitutional or local means, according to the mode of its application.

---

* We have seen premature delivery brought on by a very moderate dose of calomel and rhubarb: and abortion, by employment of diuretics, particularly the *lytta*. Hence medical men should be very cautious in administering these substances to pregnant females. EDIT.

Infliction of blows upon the abdominal or lumbar regions, or other external violence, and the introduction of sharp instruments from the vagina into the uterus, whereby the membranes of the ovum are ruptured, constitute the local and mechanical means usually employed to procure abortion. The former acts by inducing detachment of the placenta, hæmorrhage from the uterine vessels, and the death of the fœtus. But the latter mean is the most certain in its effects, and, repugnant as it may seem to every feeling of the human heart, most commonly employed. As this dreadful crime, however, is usually perpetrated by an ignorant and inexpert operator, the body of the fœtus, or generative organs of the mother, must often exhibit such marks of violence as will lead to its detection, and the punishment of the wretch by whom not only the child, but sometimes its ill-fated mother also, has been remorselessly sacrificed.*

The *signs*, denoting that abortion has taken place, are external and internal; respectively appreciable by common examination and anatomical inspection. *External.* These may be distinguished as occurring either anteriorly or subsequently, to the period of abortion. The anterior signs are cessation of the menstrual flux, depraved appetite, vomiting, protuberant abdomen, and all the other phenomena which denote pregnancy: the subsequent, commonly, are the following: issue of a watery or bloody kind of milk from the mammæ (when the woman survives the process;) sudden subsidence and diminution of these glands; areolæ of the nipples, unusually large and dark; discharge of an ichorous blood from the vagina, sometimes mixed with coagula and mucus; labia soft, red, and swollen; vagina excessively dilated; os uteri gaping, flattened, and inclining downwards; abdominal integuments wrinkled and flaccid; sometimes a disagreeable smell. The woman, moreover, feels pains about the uterine region, shivering and tremors in the extremities, and lassitude. She evinces a frequent wish to lie down, and totters in her walk. The legs sometimes swell; the superficial veins disappear; and the external parts are discoloured. In addition, it should be remembered, that the hæmorrhage which succeeds abortion or delivery, is more profuse and of longer duration than the menstrual discharge; and that, unlike the latter in its healthy state, the hæmorrhage produces great languor and exhaustion, and forms coagula, rarely or never seen in the menstrual blood. These signs, when presenting themselves in combination, assume a very decisive character; but isolately observed, their evidence must be little relied on, as most of them may be consequent upon the various other diseases to which the human female is subject. *Internal signs.* These, when the death of the mother admits of the necessary examination, are unusual capacity of the uterus, and thickness of its parietes; traces of adhesion of the placenta to the uneven internal surface of the organ; relaxation of the cervix; dilatation of the vagina; considerable diminution of the ligamenta lata; and an appearance, in the ovaries, of the cicatrix of a *corpus luteum.* But it has been contended by some gentlemen, whose arguments are perhaps more ingenious than solid, that, in any sus-

* See *The remarkable Trial of William Pizzy and Mary Codd,* &c. 8vo. Ipswich, 1808: or a very able Review of it in the sixth volume of the *Edinburgh Medical and Surgical Journal.* EDIT.

pected case, "the distention of the uterus might arise from hydatids, or moles, and the inequality of its internal surface (be) occasioned by their attachment."* And, again, it is asserted by practitioners of high authority that traces of *corpora lutea* sometimes exist in virgins, and in salacious women who have never been impregnated. All these phænomena, it should be recollected, external and internal, will be observed, whether the abortion or premature delivery have been spontaneous, or violently and voluntarily provoked; and will be more strongly marked, in proportion as the work of utero-gestation draws nearer to its close. Indeed, during its earlier stages, the traces of them are with difficulty recognizable; as the hæmorrhage, under these circumstances, is rarely profuse, and the uterus and abdomen but little distended. However distinctly visible they may have at first been, these signs commonly disappear in about ten days after the event with which they are connected has taken place.

We have before mentioned, that all these signs, taken isolately, may be fallacious, and that they only derive solidity from combination and perfect coincidence. Some objections to implicit reliance upon the results of anatomical examination have been already stated. It may be right to consider farther the identity of some of these signs with the phænomena of other and very different diseases.

In the first place, *the wrinkling and flaccidity of the abdominal integuments* may be the consequence of peritonæal dropsy, of long continued tympanitis, of tumours seated in the hypogastric or umbilical regions, or of other causes totally unconnected with abortion.

Secondly. *Secretion of milk* may arise from mere suppression of the menstrual discharge; but the fluid, in this case, will be more watery, and the mammæ less pendulous and flaccid, than after abortion.

Thirdly. All the symptoms of pregnancy may result from an imperforate state of the hymen, and consequent retention and accumulation of the menstrual blood; and all the external phænomena, characterizing abortion, from rupture of the membrane and evacuation of the retained fluid.

Fourthly. Nothing can be more uncertain than the inferences drawn from the *condition of the uterine orifice.* In some young women, the size of this orifice is naturally as large as in women who have been recently delivered: or the alterations which it exhibits may depend on some organic lesion, or other cause very different from parturition. On the other hand, it must be allowed that, in persons who are pregnant for the first time and have suffered abortion at an *early* period of utero-gestation, the wrinkling and flaccidity of the skin of the abdomen does not take place;—that, in

* See "*The Trial of Charles Angus, Esq. for the Murder of Margaret Burns.*" 8vo. Liverpool, 1808; and the two pamphlets therewith connected. A very full account of them is given in the fifth volume of the Edinburgh Med. and Surg. Journal. The writer of that article very judiciously remarks, that, "in cases of doubtful parturition, the proofs are rather to be expected in the appendages of the uterus, in the ovaries, Fallopian tubes, vagina, and areola of the nipples, than in the uterus itself. The appearances in the uterus may be the effect of disease; but such disease never produces those changes in other parts, which are the constant concomitants of pregnancy." See also *Dr. Male's Epitome of Forensic Medicine,* page 120.

some females, especially those who have been nurses, the mammæ very tardily display the changes operated by gestation;* that the destruction of the fœtus *in utero* may be accomplished without the ordinary symptoms of abortion being developed, as when, the membranes having been penetrated, and yet the placenta not detached, the fœtus perishes, and its membranes, shrinking, separate gradually from the internal surface of the womb without producing hæmorrhage; and, lastly, that the uterine orifice sometimes preserves after delivery its previous regularity of figure, or even exhibits an additional degree of straitness and constriction.

Abortion, we shall observe in concluding this part of our subject, does not always immediately succeed the infliction of the violence by which it has been determined. Sometimes the causes by which the placenta is detached, do not suffice to expel the fœtus and secundines from the cavity of the uterus. Hæmorrhage necessarily ensues; but the constriction of the cervix uteri, while it allows the issue of the blood, prevents the escape of the solid and voluminous ovum. Thus the unadhering fœtus may be retained in the womb until the hæmorrhage has completely ceased, and that enfeebled organ re-acquired its power of contraction; and, although detached by violent means, be at last expelled without any attendant hæmorrhage. To discover the truth, under such circumstances, when there is reason to suspect provoked abortion, we must have recourse to the anterior signs, and to those which succeed the hæmorrhage. If it have been but a simple loss of blood, the health of the woman will be re-established in its cessation. The detached ovum, on the contrary, acting as a foreign body, will prove a constant source of irritation to the uterus, until expelled from its cavity. The subsidence of the abdomen, the softness and flaccidity of the mammæ, the fainting without apparent cause and transient shiverings, experienced by the woman, and discharge of black fœtid substances from the vagina, indicate sufficiently the presence of a detached and putrescent body within: and this indication will be eventually confirmed by the evacuation of the putrid mass from which the train of symptoms has originated.

We now proceed to examine the queries proposed in the commencement of this article, and to indicate the points, by attention to which their solution will be best undertaken and most effectually accomplished. In so doing, we shall deviate some little from our former arrangement.

First. *Has the fœtus been prematurely expelled, or, in other words, has abortion, or premature delivery, taken place?* To decide this question, it is absolutely requisite that the expelled substance be submitted to examination; for this constitutes the *corpus delicti;* in the absence of which all judicial proceedings fall to the ground; even although the woman suspected should avow her pregnancy, and evident traces of recent parturition be discovered upon her. This examination will prove more difficult and uncertain in proportion as pregnancy is less advanced. In fact, as Foderé very judiciously observes, juridical researches on abortion can scarcely be exercised before the close of the second month of utero-gestation, both because

* Foderé has observed the mammæ of pregnant women "perfectly flaccid" between the fourth and fifth month. See his *Traité de Médecine Légale.* Tome iv.

women, at first uncertain with respect to their situation, rarely attempt to procure abortion before this period; and till then it is very difficult to collect an assemblage of signs sufficiently well-marked, in proof of its occurrence.*

We deem it needless to trace with all the minuteness and prolixity of description of the continental writers, the progressive development of the various parts and organs of the human fœtus from the moment at which its slender rudiments first become visible to its entry upon a new and independent state of existence. It will be sufficient for our purpose to state that, observed between the fourth and sixth week from conception, the fœtus appears to be about the size of a wasp. The head forms then more than half its bulk; the eyes and mouth are well-marked; the hands and feet seem to be immediately attached to the trunk; the arms, legs, and thighs are scarcely visible. The containing ovum, about the seventh week, equals in volume a large hen's-egg. This ovum (independently of the double decidua by which it is invested as the head by a double night-cap) consists of two membranes; the external, named chorion, of a spongy structure and furnished outwardly with a very thick down; and the internal or amnios, thin, transparent, and displaying amid the limpid water which it contains, the body of the fœtus. These membranes adhere less intimately together at the commencement of pregnancy, than the chorion to the uterus. Hence, we sometimes see them disunited in early abortion and separately discharged. The chorion then frequently lodges on the orifice of the womb; and the amnios, inclosing the waters and fœtus, comes away entire; while some time elapses ere the former is expelled. In this case, the woman voids a kind of membranous ovum, which has not the least appearance of flocculi; and the chorion, when afterwards discharged, unless it be attentively examined, may be mistaken for a coagulum of blood, inasmuch as it is covered by a layer of this fluid.† From this period, the numerous and intimate connexions of the chorion with the internal surface of the womb; the decided formation of the placenta; and the close relations of the fœtus with its membranes by means of the umbilical cord, render the traces of abortion, and the presence of the expelled ovum amid the accompanying coagula of blood, much less difficult of detection. In fact, the flocculi which have been described as projecting from the external surface of the chorion, soon become a thick, bloody mass, intimately attached to the uterus, and embracing the ovum in the greater part of its extent. If this mass be examined in a state of detachment from the uterus, we readily observe the bloody portion, more extensive in an inverse ratio to the term of pregnancy, embracing a vesicle that contains the fluid in which the embryo floats, not exactly at the centre, but towards one of the extremities of the mass.‡

The following constitute the chief external signs by which the prematurely delivered may be distinguished from the mature fœtus.

*First.* The body of the former is emaciated and dry; the skin, flaccid and loose, is nearly or altogether destitute of adipose substance: the blood, seen through the cutis, gives it a red or even purple colour, especially where

---

* Foderé. Work before cited. † Baudeloque. *L'art des Accouchemens.*

‡ Foderé. Work before quoted.

it is more delicately organized, as in the palms of the hands and soles of the feet. *Secondly*. The small hairs which cover the skin of the mature fœtus, are scarcely visible: the sebaceous matter of the surface is less adherent, and resembles a flocculent down, which occupies particularly the lateral parts of the face, the back, loins, and shoulders. *Thirdly*. The fontanelles are generally very large and the cranial bones mobile; but to the first of these characters there exist some exceptions. *Fourthly*. The face is little developed, and exhibits an aged, unpleasing, sorrowful appearance; the lips and ears are purple; the tongue very red. *Fifthly*. The hair is thin, short, white or silvery; the nails scarcely apparent or totally wanting. *Sixthly*. The eye-lashes and eye-brows are scanty; the lids adhering together; the pupils covered by a membrane. *Lastly*. The scrotum is purple, much wrinkled, and commonly without the testes: the labia pudendi, in the female, are swollen; the nipples not larger than a pin's-head, and destitute of the wonted areola.*

The dimensions and weight of the fœtus are too variable to admit of precise specification. In general, the full-grown child weighs from seven to upwards of eight pounds; and measures, in length, from nineteen to twenty-two inches.

These data it will be right to bear in mind; but they are applicable only in combination with all the other signs. In proportion as utero-gestation is less advanced, and as a longer space of time has elapsed from the expulsion of the fœtus, the difficulties and uncertainty of the examination of the woman must necessarily be increased. On these occasions, it is very necessary to inspect the linen of the accused; and the appearance of blood and coagula thereon will fully justify prosecution of the research. The assertion, often brought forward by women, that such appearances have been caused by a copious return of the suppressed menstrual flux, although sometimes correct, should never be implicitly received by the juridical physician, or considered as rendering unnecessary a farther investigation.

Moles, or unorganized masses, inducing all the usual phænomena of pregnancy and parturition, are sometimes discharged from the uterus; but in these, the bloody portion, which the human ovum exhibits externally, is commonly found interiorly placed, and enveloped in a strong false membrane, the production of the albuminous part of the blood. This may generally be regarded as a consequence of impregnation; and when occurring in an unmarried female, might tarnish her character, or even implicate her in horrible suspicions of secret delivery and infanticide, although it would not endanger her life: for the English law requires, that "the body of the child be found before the coroner can hold an inquest." In such cases, a mark, resembling that of the placental attachment, would be seen on examination of the uterus. The very age of a person, in whom a flooding, which assumes the characters of abortion, may have taken place, will sometimes suffice to remove every rational suspicion of such an event.†

II. *Supposing the fact of abortion to be proved, has it been spontaneous, or voluntarily and criminally provoked?* In solving this momentous question,

* Marc. *Dictionnaire des Sciences Medicales.*

† See Male and Foderé. *Works before quoted.*

the confrontation of the moral circumstances with the physical effects of the action, can alone lead to a kind of certainty; and, although such research falls rather within the province of the jury than the physician, it, nevertheless, constitutes the only mean whereby he is enabled to dissipate the obscurity which envelops the subject. The truth of this principle will be demonstrated by examination of the following points. They consist in determining whether the suspected female have suffered any fall, or other injury, followed by abdominal commotion; whether the abdomen present any bruises, or the fœtus any brown or livid spots; whether the injury were directly or shortly succeeded by hæmorrhage or other sign of disorder; whether the woman were of a temperament so irritable as to favour the operation of such violence; whether she have carried any burden, or made other effort injurious to the state of pregnancy, and at what period of this state, and what distance of time from the expulsion; whether there exist any internal cause capable of producing hæmorrhage, and whether it and the abortion have been preceded by a decidedly plethoric condition, or inordinate corporeal exercise; whether the woman have unduly compressed the abdomen with stays or ligatures, or, with a view of re-exciting the menstrual flux, have employed medicinal remedies; at what time the hæmorrhage began, its duration and probable amount; and, lastly, whether the woman exhibit any irregularity or morbid condition?

The affirmative of a majority of these queries would exclude all idea of provoked abortion; which, on the contrary, would be sanctioned by concurrence of the following circumstances:—Has the woman, in question, concealed her pregnancy? Has she sought information on the various means of procuring abortion; or used, without necessity, violent and dangerous exercise? While in the enjoyment of good health, has she been busied with preparations and arrangements indicating an expectation of illness? Has she repeatedly, and in a private manner, requested different surgeons to bleed her, particularly in the foot, without informing them of her having frequently been bled before; or, has she sought to obtain from any medical practitioner, or other person, articles proper for provoking hæmorrhage? Has she elsewhere procured this kind of drugs, or employed compounds of them prepared by herself? Has she, needlessly and without the prescription of a medical adviser, taken drastic purgatives; or have the remains of suspected drugs been found in her possession? Has she suddenly affected illness in order to conceal her state, and especially the uterine hæmorrhage subsequently discovered. Do there, lastly, exist on the generative organs of the mother, or body of the fœtus, marks indicating the direct infliction of violence by blows, or the puncture or laceration of sharp instruments, introduced by the vagina? All these, and other analogous circumstances, or the greater part of them, being combined, announce premeditation, and invest with an undoubted criminal character, the fact of abortion. It ought, however, never to be forgotten that, certain direct operations excepted, most of the other remedies and circumstances may have been employed, or have taken place, without the most distant intention, on the part of the woman, to procure abortion. Of this we have witnessed several unquestionable instances.

III. *Was the fœtus, at the period of employment of the expulsive means,*

*endued with life?* In resolving this last query, we shall refrain from all idle discussions respecting the period of animation of the fœtus; for the existence of organic life alone establishes vitality, and this dates from the moment of conception. It is here absolutely requisite that the body be examined: and, if this present depression of the fontanelles without traces of external violence, separation of the epidermis in many places, livid colour of the skin, a cadaverous smell, an almost pulpy softening of the muscles and viscera; a shrivelled, tender, fœtid, condition of the umbilical cord; a puffiness of the surface of the body, especially of the face and abdomen; we may conclude that the fœtus has perished anteriorly to its expulsion, without, however, being able to indicate precisely the period of death, or establish with certainty, in all cases, whether it have been determined by the employment of any peculiar means. Thus, for instance, if abortives have been used on the days just preceding abortion, and the mother have not felt the motions of her child for some time previously to such application, prudence and humanity would alike suggest the most favourable construction.—From what has been just said, it results, that the resolution of our third query is only practicable when the space of time, which has elapsed from expulsion to examination of the fœtus, is not sufficiently considerable to admit the supposition of extra-uterine putrefaction. The development of this process is variable, and can only be appreciated, in an approximative manner, by a comparison of different circumstances.

After the view already taken of this subject in its physiological and physical aspects, it now remains for us to survey it as connected with Jurisprudence. In so doing, we shall cursorily examine the light in which destruction of the fœtus has been regarded by some of the more enlightened nations, and in different ages, of the world; the degree of criminality attached to the premeditated act; and of penal severity with which its perpetration has been denounced or visited. In fact, we shall trace the judicial history of procured abortion from a remote æra to the present time; and terminate the rapid sketch by an exposition of the state of legislation respecting the crime, as it now exists among us. To the influence which sound ethics, and the adoption of a vigilant and comprehensive system of jurisprudence may exert in preventing, or rendering less frequent, this outrage upon the best interests of society, moral and political, we shall also occasionally advert.

1. *Opinions of the Ancients respecting provoked Abortion;* and Retrospect of Legislation, as it regards this crime, to the commencement of the present Century.* The opinions and practice of some of the more polished nations of antiquity with respect to voluntary abortion, constitute a feature, singular as dark, in the history of the human mind. The passages which, in the

---

* By medical writers, the term Abortion is generally restricted to expulsion of the human ovum before the close of the sixth month of utero-gestation: and that of premature labour to untimely parturition after that period. In juridical medicine, this distinction is destitute of utility. Laceration of the perinæum, we had nearly forgotten to mention as one of the signs of premature delivery. That it will not be observed in cases of abortion, or discharge of uterine hydatids, every one will be aware. We may farther remark, that clavus or the ergot of commencement of this article, we have quoted from the Roman poets, incontestibly prove that destruction of the human embryo was not restricted to the tent of the wandering savage, or exclusively practised upon the fruit of illicit love. By the high-born matrons of imperial Rome, in the meridian of her greatness and illumination, the most sacred claims on the female heart were remorsely violated, and the laws and interests of society, broken and sacrificed with shameless impunity. Some of the more celebrated philosophers of Greece had even recommended the employment of abortion as a mean whereby the equilibrium of population might be preserved; and the eloquent Cicero, in alluding to the case of a woman who had been condemned for destruction of her offspring, founded the justice of this decision rather upon the civil consequences, than on the moral turpitude of the crime.*

To what source shall we trace the introduction of these barbarous and unnatural prejudices among the enlightened nations of antiquity? On reverting to the civil history of these remote times, it will be found that abortion was regarded criminal or otherwise according to the doctrines of the prevailing sect of philosophy: and, by some of these, the embryo or fœtus was described as not animated by a rational and thinking soul. Now, of all the opinions of philosophers, those of the Stoics seem to have been most generally adopted by the Roman legislators: and, with an affectation of indifference peculiar to their sect, the disciples of Zeno regarded the union of soul and body as only first taking place in the act of respiration. Consequently, the fœtus, so long as it remained in utero, could not be endowed with a thinking soul. Applying, therefore, this abstruse and erroneous principle to criminal legislation, they utterly divested voluntary abortion of its atrocious character: and alike refused to the fœtus the title of a human being and the protection of human laws. It was merely named *Pars viscerum matris*, a portion of the viscera of the mother.† Thus, by metaphysical subtleties, wholly destitute of foundation in reason and experience, were the conquerors of the world governed; and thus was favoured the introduction of licentious morals, already too prevalent in the earlier ages of the Roman empire. Ideas, equally inaccurate, respecting the animation of the fœtus, probably exist among the uncivilized nations by which, at the present day, the employment of abortives is openly allowed or tolerated:

---

rye has been lately recommended as a remedy which may be exhibited with powerful effect to expedite the birth of the child in lingering labour, or to obviate retention of the placenta. In addition to the property which this curious vegetable substance possesses of stimulating the gravid uterus, it is said to operate with considerable certainty in restoring the suppressed menstrual discharge. *Bigelow, on the Use of Clavus, or the Ergot of Rye. New England Journal of Medicine and Surgery.* Vol. v. No. 2. See also *Journal of Science and the Arts.* No. III.

* Memoriâ teneo quamdam mulierem, cum essem in Asia, quòd, ab hæredibus secundis accepta pecunia, partum sibi ipsa medicamentis abegisset, rei capitalis, esse damnatam; neque injuria; quæ spem parentis, memoriam nominis, subsidium generis, hæredem familiæ, *designatum reipublicæ civem*, sustulisset. *Cicero. Oratio pro A. Cluent. Avito.*

† By the laws of Scotland, this obviously absurd principle is retained to the present day.

or, like the women of the island of Formosa, they are impelled to the act by certain religious principles which exclude all thought or imputation of crime.

To the same pernicious direction of the human mind may be principally attributed the occasional prevalence of destruction and exposure of infants in different regions of the globe. Warlike and republican people, haunted by the groundless dread of excessive population, looked upon child-murder as a necessary evil. Aristotle even declared his opinion that, in a republic, the number of citizens should be restricted, and the education of illegitimate offspring proscribed: and by Lycurgus, this horrible precept was transformed into a law. Strabo reports, that the inhabitants of Cathea subjected their children, at the age of two months, to the inspection of a magistrate, who selected the more robust for preservation, and condemned the rest to perish. A practice, somewhat similar, was adopted by the original founders of Rome. The Celts exposed their new-born infants upon a shield to the current of a river, and considered as legitimate only those which survived this perilous ordeal. From such revolting scenes of darkness and barbarism, it is cheering to turn and contemplate a nation distinguished by its humanity. The Thebans, a people who kept prostitutes in their temples, punished with death the exposure of infants; and provided, by a special law, for the education of those children whose parents were incapable of supporting them.

By the Hebrew law, heavy penalties were denounced against the crime of procured abortion, and even death, if the mother fell a martyr to its consequences.* Constantine the Great not only directed the towns of Italy and Africa to assist those who declared themselves unable to educate their children: he instituted the most dreadful punishment against destruction of the offspring.† Under the Emperors Severus and Antoninus, the absurd doctrine had been introduced that the fœtus only became animated at a certain period of utero-gestation. Hence, the distinction of animated and inanimate fœtus was set up: and, while infliction of an extraordinary penalty was deemed sufficient to expiate destruction of the latter, the murderer of the animated fœtus incurred the utmost vengeance of the law. But Christianity, which had operated such great changes in the legislation of Rome, exercised an equal influence on every thing relating to her morals. The successors of Constantine denounced with severity the crime of abortion; nor even admitted a distinction as to the rectitude or malignity of design with which the remedies, provoking it, had been administered. Renouncing the opinions of the schools, many fathers of the church observed, that the fœtus, formless as it appears, must be animated, since it grows; and adopted a maxim, alike salutary in its moral and political operation: *Homo est qui futurus est.* Thus the Congress, assembled in Constantinople in 692, ordained that the crime of abortion should be punished with the same severity as homicide. Yet, either from a feeling of indulgence to human frailty, or from the dominion which the doctrines of Plato long held over

---

* Exodus. Chapter xxi, verses 22—23.

† Neque gladio subjugetur, sed insutus culeo, et inter ejus furales angustias comprehensus, serpentum contuberniis misceatur, ut omni elementorum usu vivus carere incipiat, ut cœlum superstiti, terra mortuo auferatur. Cod. Theodos. C. ix. tit. 15.

the fathers, the canon law was less rigorous than the Roman law, as reformed by the Emperors of the East: for by the distinction between the quick and inanimate fœtus, to which we have already alluded, the magnitude of the crime, and severity of the punishment, were judged and regulated. This distinction yet constitutes a feature in the legislation of several countries. We scarcely need observe, that it is not less erroneous in principle, than hostile in practice to sound morality, and the vital interests of the social system.

By France, as by some other nations, the reformed Roman law was adopted in all its rigor. Midwives, and in general, all medical practitioners, convicted of the crime of provoked abortion, received sentence of death. An edict of Henry II. confirmed by succeeding sovereigns, established little difference between the criminality of the female who concealed her pregnancy, or the wretch by whom a fœtus was secreted or destroyed. And letters of pardon were even sometimes requisite for the security of persons, who, ignorantly and without criminal design, had employed remedies capable of determining abortion, or been compelled to resort to this formidable expedient with a view of rescuing the mother from some more urgent peril. But the inflexible and undiscriminating spirit of the laws of Draco, which so long influenced the criminal legislation of every country where the Roman law was received, has ceased to operate. By the penal code instituted in the first years of the French republic, the crime of infanticide was made punishable by death, and that of provoked abortion, by twenty years imprisonment. "Life, in the contemplation of the *English law*, begins as soon as a child is able to stir in its mother's womb; for, if a woman be quick with child, and any one by a potion or otherwise, killeth it in her womb, or beat her, whereby the child dieth in her body, and she is delivered of a dead child, this, though not murder, was by the ancient law, homicide; but the modern law does not look upon this offence in quite so atrocious a light, but merely a heinous misdemeanour; yet if the child be born alive, and afterwards die in consequence of the potion or beating, it will be murder."*

II. *Existing Legislation of the French and English Criminal Courts, with regard to the Crime of Abortion.* The French code of 1810, "more comprehensive, and better adapted to the nature of civilized man," than its predecessor, decrees, that "whoever, by food, potion, medicine, violence, or any other mean, shall have procured the abortion of a pregnant woman,

---

* See *Blackstone's Commentaries*. Vol. i. p. 129; or Male's *Epitome of Forensic Medicine,* p. 113. We wish to restrict as much as possible the limits of this historical sketch. It may, therefore, suffice cursorily to observe, that, among the ancient inhabitants of Germany and Gaul, the person guilty of procuring abortion was allowed to expiate the offence by the payment of a sum of money; that, by the congress of Ancyra, in 314; of Lerida, in 524; and of Mayence, in 847; long penance, with exclusion from the Sacrament, was decreed against the crime; and that capital punishment, eternal irregularity and excommunication, were respectively denounced, by the Roman Pontiffs, in 1588 and 1591, against the principal culprit, and every priest and layman, implicated as accomplices in the charge. Sextus Quintus reserved to himself the exclusive right of absolution. By Gregory this privilege was granted to every ecclesiastic.

either with her consent or otherwise, shall be punished with imprisonment;"—that "the same punishment shall be denounced against the woman who shall have procured abortion in herself, or consented to employ means prescribed or administered to her for such purpose, if abortion have been the consequence of them;" and, lastly, that "physicians, surgeons, and other officers of health (officiers de santé), as well as apothecaries, who shall have prescribed or administered these means, shall, in the event of abortion taking place, be condemned to hard labour for a certain time."* Finally, *the English law*, as it is at present framed, declares, that, "if any person, after the year 1803, shall wilfully and maliciously administer to, or cause to be administered to, or take any medicine, drug, or other substance or thing whatsoever, or use, or caused to be used or employed, any instrument, &c. with intent to procure the miscarriage of any woman, not being, or not being proved to be, quick with child at the time of committing such thing or using such means, then, and in every such case, the persons so offending, their counsellors, aiders, and abettors, shall be, and are declared guilty of felony, and shall be liable to be fined, imprisoned, set in and upon the pillory, publicly or privately whipped, or transported beyond the seas for any term not exceeding fourteen years." By the same Act it is ordained, "that administering medicines, drugs, &c. or using means with the intent to procure abortion, *after quickening*, shall be punishable with death."†

The crime of voluntary abortion, comprehensively examined, presents some important views, upon which, although not immediately interesting to the physician in the exercise of his juridical functions, we are anxious, ere concluding this article, to direct the attention of the moral and political philosopher. Seduced by the attractions of the subject, we have already transgressed the limits originally prescribed to ourselves in discussing it; but the subject itself may, we hope, procure from the reader that indulgence and prolonged attention which our desultory and imperfect style of discussion will not be found to merit or excite.

What is the nature of the phænomenon termed quickening? and does it afford any rational ground of distinction with respect to the period of animation of the fœtus? Commonly, between the sixteenth and twentieth week from impregnation, the woman becomes sensible of a fluttering motion, sometimes visible even at a distance, in the lower part of the abdomen; and often preceded, or accompanied, by a sensation of flatulence and faintness. This is called quickening, as supposed to denote the first movement, hence to constitute the earliest sign of animation, of the fœtus. But by some physiologists different, and, in our opinion, more rational explanations of this phænomenon have been attempted.‡ And if on the one hand, the axiom which we have adopted in the former part of this article be correct, that "the existence of organic life alone establishes vitality;" and if

---

* See *Code Penal* (of France), § 317; *Foderé Traité de Médecine Legale*, tom. IV. p. 386; and *Dictionnaire des Sciences Medicales*, tom. ii. *Article, Avortement.*

† See *Statutes at Large*, 43 Geo. III. cap. 28, and *Dr. Male's Epitome*, p. 114.

‡ Some late writers, in one of our periodical Journals of Medicine, (we forget which), has given an ingenious explanation of the phenomena of quickening. The unimpregnated uterus, it is well known, lies completely within the cavity the doctrine of the venerable fathers, on the other, be undeniable in fact, as comprehensive and beneficent in operation; the distinction of inanimate and animated fœtus, introduced into the legislation of Rome, under the auspices of her emperors, and subsequently engrafted upon that of other European countries, should be utterly rejected.* Sophistry, indeed, may affect to trace, with subtle finger, the boundary which separates the crime of voluntary abortion before the term of quickening from those of destruction of the fœtus at a more mature period, and of infanticide. The unerring voice of truth and nature disclaims its acknowledgment. In fact, to the eye of reflection, child-murder, and destruction of the shapeless embryo, present a character equally atrocious; and in their influence upon the moral and physical constitution of human society, inflict a wound equally deep and malignant. Opinions, like these, cannot be too often or too strenuously inculcated on the public mind. Whether doctrines, wholly unsound in principle, can be consistently retained in practice, forms a query, which it is the province of men more deeply versed than ourselves in the science of legislation to agitate and resolve. In medicine, the solution of such a problem would require but little deliberation.

Yet, should we be disposed to deprecate any law, which, like the unsparing edict of Henry the Second of France, might regard, as equally criminal, the concealment of pregnancy and destruction of the offspring, and punish, with indiscriminate vengeance, the unhappy victim of seduction, and the monster reeking from the premedited sacrifice of her child. A law, indeed, thus worthy of the genius of Draco, must have been founded on very superficial and inaccurate views of the female character; and will, we hope, never again disgrace an European code of legislation. Many an unfortunate woman will shrink from a humiliating confession of her shame, in the vague hope that death may intervene to conceal her wretchedness and dishonour; who would reject with disdain the proffered instrument of abortion, and rather endure the extremes of misery and neglect, than raise the weapon of destruction against the life of her offspring.

But as no dread of penal consequences can ever operate to the utter suppression of the crime, the views of a wise legislature should be directed, rather to the means of prevention than those of punishment. It would be foreign to our subject to enter formally upon this comprehensive field of discussion. We shall content ourselves with throwing out a few hints upon the means best calculated to diminish the frequency of voluntary

---

formed by the pelvic bones. Impregnation having taken place, the organ gradually enlarges, and the time must obviously come when it can no longer be confined within such narrow limits. Arrived at a certain point of expansion, the uterus at length suddenly emerges from the pelvis into the abdomen; and thus, contends the gentleman in question, the motion, and all the train of sensations attendant on quickening, are produced.

* By some of the ancient legislators, the period of animation of the fœtus has been fixed at forty days; by some at seven days; by others at eighty. The fact is, vitality dates from the first stroke of the *punctum* saliens, and the future development of the fœtus is steadily progressive. Hence the rude and mis-shapen embryo has the same relations with society, the same claim upon the protection of its laws, as the more mature fœtus. EDIT.

abortion and infanticide. They will admit of separate consideration, as exercising a moral operation, or coming within the department of medical police.

Whether pregnancy be the consequence of legitimate union, or illicit intercourse, it presents an equal claim upon the interest of society. This principle, however neglected in times of ignorance and barbarism, is now acknowledged in our systems of legislation. Yet public opinion inflexibly retains its ancient severity, and continues uninfluenced by the mild and indulgent temper of modern institutions, which that principle has served to inspire. We are far from considering faulty that direction of ideas by which purity of morals, and the maintenance of social order, are promoted and secured: we wish not to see the dishonoured female and the virtuous wife placed in the same rank, and attracting equally the consideration of society. Against that rigid intolerance of the public mind we protest, which loads with exclusive infamy the victim of seduction; while the more guilty author of her wretchedness, unmolested by the execration which he merits, and little if at all degraded in public estimation, pursues elsewhere his infamous career. Some French writers have gone so far as to declare, that those countries, in which the laws of chastity are most severe, and their violation is most heavily fraught with dishonour, exhibit more frequently than others the crime of child murder and abortion.* We shall refrain from expressing our own opinions on this subject, lest, while advocating the cause of the injured and destitute female, and urging her potent claims to mercy and commiseration, we should be suspected of a wish to sap the foundations of morality, and set up ourselves as the apologists of profligacy and crime. Nothing is more distant from our intention. How far the exertions of the legislature to facilitate and promote marriage among the inferior classes of society, by conferring peculiar privileges and exemptions on the wedded state, might operate in repressing the dreadful crime of fœticide, is a query more readily proposed than decided.

We have, secondly, to consider the influence which a vigilant system of jurisprudence may exercise in preventing the frequency of this inhuman practice. The waste of human life, resulting from voluntary abortion, or from attempts to provoke it, is, we are convinced, far greater than they whose attention has never been directed to the subject, or possess not the requisite means of calculation, will be disposed to credit. There is scarcely a town or village in the kingdom where this horrible traffic is not carried on with fearless impunity, and the uncouth instrument of some mercenary wretch occasionally employed to bring about what the midwife or emmenagogue of the druggist has previously failed to accomplish. Many young women, the victims of obscure and violent disease, annually perish from this unsuspected cause. Any thing like a remedy for this deep and insidious evil, it is in the power of Legislature alone to furnish and apply. The Act which we have last quoted, is inadequate to its suppression. Under the specious title of emmenagogues, powerful preparations of the hydrargyrus, lytta, and other violent intestinal stimulants, are yet prescribed or administered with little hazard or precaution. A law, which, rigorously

* *Dictionnaire des Sciences Médicales,* tome ii. *Article, Avortement.*

enforced, should prohibit, under severe penalties, all persons from prescribing, administering, or employing any remedy, medicinal, dietetic, or mechanical, in cases of suppressed menstruation or suspected pregnancy, except by the advice or with the sanction of a regularly educated physician or surgeon, would probably go far to restrict the operation of the evil, and diminish the mortality consequent on it. That any British practitioner of medicine could so far forget the object of his art, or pollute the dignity of the professional character, as to undertake, from mercenary considerations, the work of assassination, is incredible: we will not dishonour our country by admitting the supposition.

An important question, relative to the subject of abortion, lastly, presents itself for our consideration. In extreme cases of malformation of the female pelvis, where delivery of the fœtus at the full term is obviously and utterly impracticable, may abortion or premature delivery be justifiably induced, and thus the life of the child be sacrificed to the preservation of the mother? This point has been long and warmly contested. The respectable and conscientious Mahon formally protests against the practice; but his arguments are ably refuted by Foderé: and most of our readers will, we imagine, concur in the opinion of Dr. Male, that it is "both legally and morally right rather to save a certain and valuable life than risk it for an uncertain one;" and that the propriety of the expedient is alike "sanctioned by humanity, policy, and justice." Nor should it be forgotten, that, in such cases, the life of the infant is not invariably sacrificed; for it sometimes, though rarely, happens, that a fœtus, born before the seventh, and even at the fifth month of utero-gestation, will live and ultimately do well. Several such cases, most respectably attested, are upon record.*

---

### XIV.

### REVIEW OF THE MEDICAL DEPARTMENT OF THE EAST INDIA COMPANY.

FOR several years past we have witnessed, with pain, the depressed state of the medical profession in this country. The ranks of medical society are so crowded, that the professors of a noble art are driven, by actual want, to the most disingenuous devices—to the necessity of stabbing one

---

* A case of this kind is cited by Mahon from Brouzet. See also a case by Dr. Rodman, Edinburgh Medical and Surgical Journal, vol. xi. p. 455. We take this opportunity of calling the attention of the reader to an error which we have discovered in the commencement of this article, and which, however obvious, we think it right to notice. In describing the *internal signs* of pregnancy and parturition, we mentioned, that they will be more strongly marked in proportion as utero-gestation is farther advanced, and will disappear shortly after delivery; but from this description the *corpora lutea of the ovaries* should be excluded, as they generally remain visible during life.

